# Disitamab vedotin in combination with immune checkpoint inhibitors for locally and locally advanced bladder urothelial carcinoma: a two-center’s real-world study

**DOI:** 10.3389/fphar.2023.1230395

**Published:** 2023-08-14

**Authors:** Yongbao Wei, Ruochen Zhang, Chenbo Yu, Zhiwei Hong, Le Lin, Tao Li, Jianhui Chen

**Affiliations:** ^1^ Shengli Clinical Medical College of Fujian Medical University, Fuzhou, China; ^2^ Department of Urology, Fujian Provincial Hospital, Fuzhou, China; ^3^ Department of Urology, Fujian Medical University Union Hospital, Fuzhou, China

**Keywords:** bladder cancer, bladder-sparing protocol, RC48, PD-1, HER2, antibody-drug conjugate, immunotherapy

## Abstract

**Objective:** Our study aims to assess the effectiveness and safety profile of Disitamab Vedotin (DV, RC48-ADC), an innovative humanized anti-HER2 antibody conjugated with tubulin-disrupting antimitotic drug monomethyl auristatin E (MMAE) via a cleavable peptide linker. This treatment combined immune checkpoint inhibitors as part of the bladder sparing approach for selected patients suffering from locally and locally advanced bladder urothelial carcinoma.

**Patients and methods:** We conducted a two-center, real-world study involving locally advanced urothelial carcinoma (UC) patients. Patients were classified based on HER2 expression (IHC 3+/2+/1+) or lack of HER2 expression (IHC 0). The primary endpoint was the objective response rate (ORR), assessed by the investigator following the criteria of RECIST V1.1. Secondary endpoints encompassed the pathological complete response rate (pCR), pathological partial response rate (pPR), and pathological stable disease (pSD), along with recurrence-free survival (RFS), the pathological downstaging rate, and the safety profile of the treatment.

**Results:** In this study, nine patients were enrolled, with a median follow-up duration of 12.0 months. The overall confirmed ORR was 88.9%, Five patients achieved a complete response (CR), and three patients achieved a partial response (PR). The radiological complete response (rCR) aligned perfectly with pCR. The median radiological progression-free survival (rPFS) spanned 12.0 months (range from 8.0 to 17.0 months). One patient diagnosed with disease progression (PD) underwent a radical cystectomy. The pathological stage evolved from T2N0M0 to T3aN2M0, followed by adjuvant chemotherapy with a gemcitabine-cisplatin (GC) combination radiotherapy. At the 9-month follow-up, neither recurrence nor metastasis was observed. The rate and intensity of complications were manageable among these patients, with no evidence of grade 4 and 5 adverse events.

**Conclusion:** The combination of DV and PD-1 demonstrated considerable activity in the objective response rate (ORR) in patients with HER2 IHC 0/1+/2+/3+ muscle-invasive bladder cancer (MIBC), along with the longest reported median radiological progression-free survival (rPFS) to date. With an extended duration of treatment, the safety profile of DV plus PD-1 was also confirmed to be manageable.

## 1 Introduction

Urothelial bladder cancer (UC) ranks the ninth most common cancer worldwide, with an annual incidence of over 500,000 new cases and 200,000 deaths attributable to the disease (Bray et al., 2018). Only 30% of these newly diagnosed cases involve muscle-invasive bladder cancer (MIBC), where the tumor invades the detrusor muscle (Charlton et al., 2014). A combination of Neoadjuvant chemotherapy plus Radical cystectomy (RC) has traditionally been the standard of care for MIBC. Current RC methodologies have achieved 5-year overall survival (OS) rates ranging from 56% to 66% (Grossman et al., 2003; Sherif et al., 2004; Stein et al., 2001; Zehnder et al., 2011).

Over recent decades, there has been a growing trend toward organ-preserving therapies in treating numerous cancers. Within the sphere of bladder cancer, introducing a disciplinary approach has led to the development of bladder-sparing approaches. These approaches combine maximal transurethral resection (TURBT) with radiotherapy and concurrent radio-sensitizing chemotherapy to treat MIBC. While no definitive randomized studies have compared RC and this bladder-sparing trimodal therapy (TMT), multiple series suggest that TMT may produce favorable outcomes for carefully chosen patients (Hoskin et al., 2010; Efstathiou et al., 2012; James et al., 2012a; Mak et al., 2014).

The recent application of immune checkpoint inhibitors (ICIs) has yielded significant clinical outcomes, thereby establishing a role in treating metastatic UC (mUC) and MIBC. This includes inhibitors such as pembrolizumab, nivolumab, avelumab, tislelizumab, and toripalimab. Using neoadjuvant cisplatin-based chemotherapy (NAC) and PD-1/PD-L1-based immunotherapy has demonstrated pathologic complete response rates of approximately 30%–40% and 30%–50%, respectively, (APOLO et al., 2017; SHARMA et al., 2017; Powles et al., 2019; Guptal et al., 2020; Sheng X. et al., 2021; Necchi et al., 2019; Bellmunt et al., 2021; Ye et al., 2021; Christopher et al., 2022). Furthermore, immunotherapy has been linked to a notably high complete response rate (up to 80%) in conjunction with bladder-sparing treatment, an improvement compared to traditional TMT, which achieves 60%–70% (COPPIN et al., 1996; SAUER et al., 1998; JAMES et al., 2012b; Balar et al., 2021; Garcia delMuro et al., 2021). The observed outcomes underscore the need for additional research into the application of ICIs in combination therapies for the curative treatment of MIBC patients.

In bladder cancer, human epidermal growth factor receptor 2 (HER2) overexpression is strongly linked with tumor progression and poor prognosis, although HER2 genomic amplification is not a typical mechanism (Chow et al., 2001; Jimenez et al., 2001; Kruger et al., 2002; Fleischmann et al., 2011). Disitamab vedotin (DV, RC48-ADC) is an innovative humanized anti-HER2 antibody, conjugated with MMAE via a cleavable linker. DV has demonstrated promising antitumor activity and a tolerable safety profile toward mUC, exhibiting an objective response rate (ORR) of 51.2% and median progression-free survival (PFS) of 6.9 months (Sheng Xinan et al., 2021). In the RC48-C014 trial, 32 patients with metastatic UC were treated with DV and toripalimab, resulting in an ORR of 71.8% in the overall population and 73.9% in first-line previously untreated patients (Sheng et al., 2022). Additionally, DV combined with PD-1 inhibitors has yielded satisfactory efficacy in patients with MIBC and non-muscle Invasive Bladder Cancer (NMIBC) (Wen, 2022; Hu et al., 2023; Huang et al., 2023). Nevertheless, real-world studies, which provide crucial information on a drug’s efficacy and safety within the actual patient population, are lacking for the role of DV in MIBC. Therefore, this study aims to explore the use of DV for the treatment of locally advanced bladder urothelial carcinoma, utilizing real-world data.

## 2 Methods

### 2.1 Study design

This real-world study retrospectively analyzed the clinicopathological and follow-up results of patients with locally or locally advanced primary urothelial carcinoma of the bladder, treated with DV and immunotherapy at Fujian Provincial Hospital and the Union Hospital Affiliated with Fujian Medical University. The inclusion criteria should meet the following requirements, patients without DV or immunotherapy contraindications; ECOG score were 0 or 1; aged between 18 and 85 years; patients without underlying severe medical conditions. In addition to the following requirements, they should also meet the criteria as follows:

1. Diagnosis and treatment were managed comprehensively within the two centers.
2. Detailed clinicopathological data were available.
3. Primary urothelial cell carcinoma of the bladder was confirmed pathologically, excluding specific differentiation, such as sarcoma or clear cell.
4. The study only included locally and locally advanced UC, with the staging at cT2-T4aN0-2M0.
5. At least two or more courses of this combined neoadjuvant therapy were included.
6. The therapeutic response could be evaluated.

DV was administered according to the RC48-C014 study protocol, i.e., DV 2 mg/kg (Equivalent to dose of 1.5 mg/kg using DV-based extinction coefficient outside of China) every 2 weeks (Q2W), and the immunotherapy consisted of either tislelizumab 200 mg every 3 weeks (Q3W) or toripalimab 3 mg/kg Q2W. The study was approved by the ethics committees of both centers and received written informed consent from the patients’ families.

### 2.2 Data collection and evaluation

Patients enrolled in this study started drug combination therapy in December 2021, with follow-ups extending until July 2023. The collected data encompassed demographic information, bladder cancer history, pathological data, details of neoadjuvant drugs and course of treatment, surgical interventions, and primary and secondary study endpoints. The pathological grade was classified into Grade 1, grade 2, and Grade 3 according to WHO 1973 criteria. HER2 expression was evaluated by immunohistochemistry (IHC), with categories being IHC 0; 1+; 2+; 3+, or positive or negative as determined by the FISH gene testing. The expression of programmed cell death protein 1 (PDL-1) was evaluated and was classified as ≥1% and <1%. All patients had a pelvic Magnetic Resonance Imaging (MRI) for bladder measurable lesions in baseline before combination therapy.

Neoadjuvant immunotherapy drugs included tirelizumab, administered at 200 mg every 3 weeks (Q3W), or toripalimab, dosed as per literature at 3 mg/kg every 2 weeks (Q2W) for 12 weeks. The RECIST 1.1 standard evaluated the efficacy of neoadjuvant therapy. We assessed the imaging efficacy (categorized as rCR, rPR, rSD, and rPD) of bladder lesions in patients by MRI, and assessed pathological efficacy (categorized as pCR, pPR, and pSD) by MRI combined with bladder biopsy or TURBT, and evaluated the lungs and abdomen by Computed Tomography (CT) to assess the presence of distant metastases at a frequency of every 3–6 months.

After neoadjuvant therapy, patients underwent a second surgical treatment, including diagnostic radical transurethral resection of bladder tumor (TURBT) and/or bladder biopsy, partial cystectomy, or radical cystectomy. Secondary study endpoints such as pCR, pPR, and pSD were determined based on pathological findings. Secondary study endpoints included the rPFS in months following neoadjuvant therapy and pathological degradation. Concurrently, side effects and their severity were evaluated according to the adverse reaction classification.

## 3 Results

### 3.1 General information

We screened 53 patients, and nine patients with primary bladder urothelial carcinoma from two institutions were finally included in this study, among which the ratio of males to females was 8:1 (Table 1). All of these patients were platinum-tolerable. Still, after detailed explanation and communication, these patients accepted DV combined immunotherapy as a new treatment. These patients had a median age of 72 and a median BMI of 22.3 kg/m^2^. Eight patients had an Eastern Cooperative Oncology Group (ECOG) performance status score of 0, while one scored 1. Among the participants, six patients were newly diagnosed, and three experienced recurrences after bladder sparing treatment (two for the third time and one for the second time).

**TABLE 1:** Detailed general patient information.

	Case 1	Case 2	Case 3	Case 4	Case 5	Case 6	Case 7	Case 8	Case 9
Age (years)	70	72	63	79	60	80	84	66	73
Sex	Male	Male	Male	Male	Male	Male	Female	Male	Male
BMI (m^2^/kg)	22.3	23.3	25.4	21.3	21.2	21.6	18.7	25.7	25.3
ECOG	0	0	0	0	0	1	0	0	0
Comorbidities	0	Prostate cancer (lower risk)	Diabetes	0	0	Diabetes, Hypertension	Hypertension	Hypertension	0
Previous treatment	Diagnostic-TURBT	Diagnostic-TURBT	Diagnostic-TURBT	Diagnostic-TURBT	Diagnostic-TURBT, GC (3 cycles)	Diagnostic-TURBT	Diagnostic-TURBT, GC + BCG	Right-sided laparoscopic radical resection for ureteral cancer, GC (2 cycles)	Diagnostic-TURBT, GC + Triprilimab (6 cycles)
Initial diagnosis or Recurrence	Initial	Initial	Initial	Initial	Initial	Initial	Bladder cancer recurrence (the second time)	Bladder cancer recurrence after ureteral cancer	Bladder cancer recurrence (the second time)
Number of Lesions	More than 3	Single (>3 cm)	More than 3	More than 3	More than 3	More than 3	More than 3	More than 3	More than 3
cTNM	T2N2M0	T3bN2M0	T2N0M0	T3bN2M0	T2N0M0	T2N0M0	T3bN1M0	T4aN3M0	T4aN1M0
pTNM	T2N2M0	T3bN2M0	T2N0M0	T3bN2M0	T2N0M0	T2N0M0	T3bN1M0	T4aN3M0	T4aN1M0
Type of pathology	UC	UC	UC: Carcinoma *in situ*, micropapillary subtype	UC	UC	UC	UC	UC	UC
Pathological Grading	3	3	3	3	3	3	3	3	3
HER2 expression(IHC)	2+	2+	3+ (20%)	3+ (30%); 2+ (70%)	2+ (70%)	0	1+	2+	3+
PDL-1 expression	< 1%	< 1%	< 1%	< 1%	< 1%	< 1%	< 1%	< 1%	< 1%
Immunotherapy	tislelizumab 200 mg Q3W, two cycles	tislelizumab 200 mg Q3W, two cycles	tislelizumab 200 mg Q3W, eight cycles	toripalimab 3 mg/kg Q2W, seven cycles	tislelizumab 200 mg Q3W, three cycles	tislelizumab 200 mg Q3W, three cycles	toripalimab3mg/kg Q2W, three cycles	toripalimab 3 mg/kg Q2W, 10 cycles	toripalimab 3 mg/kg Q2W, 14 cycles
DV therapy	RC48 2 mg/kg, Q2W, two cycles	RC48 2 mg/kg, Q2W, two cycles	RC48 2 mg/kg, Q2W, eight cycles	RC48 2 mg/kg, Q2W, five cycles	RC48 2 mg/kg, Q2W, three cycles	RC48 2 mg/kg, Q2W, three cycles	RC48 2 mg/kg, Q2W, four cycles	RC48 2 mg/kg, Q2W, two cycles	RC48 2 mg/kg, Q2W, 14 cycles
ycTNM	T0N0M0	T0N0M0	T0N0M0	T0N0M0	T1N2M0	T4aN0M0	T2N0M0	T2N2M0	T1N0M0
ypTNM	T0N0M0	T0N0M0	T0N0M0	T0N0M0	T0N0M0	T3aN2M0	T2N0M0	T2N2M0	T1N0M0
Imaging efficacy assessment and results	MRI; rCR	MRI; rCR	MRI; rCR	MRI; rCR	MRI; rPR	MRI; rPD	MRI; rPR	MRI; rPR	MRI; rPR
Pathological efficacy assessment and results	pCR	pCR	pCR	pCR	pPR	pPD	pPR	pPR	pPR
Subsequent treatment	Radical cystectomy	TURBT + Biopsy	TURBT + Biopsy	Uretoscopy + Biopsy	TURBT, Partial cystectomy and dissection of pelvic lymph nodes	Radical cystectomy, GC, radiotherapy	TURBT + Biopsy, RC48 plus Teripulimab Maintenance	TURBT + Biopsy; RC48 plus Teripulimab	TURBT + Biopsy, RC48 plus Teripulimab
Follow-up (months)	12.0	13.0	14.0	12.0	8.0	9.0	9.0	17.0	15.0

All patients have pathologically diagnosed with bladder urothelial carcinoma: eight were diagnosed with diagnostic TURBT, and one was diagnosed through laparoscopic radical ureteral carcinoma resection (during which ureteral carcinoma was found to involve the bladder wall during the operation with positive incisal margin, postoperative MRI review showed bladder wall thickening and enhancement, which was considered as residual bladder cancer). Staging for all cases was T2-T4aN0-3M0, with four cases each at stages T2 and T3. All pathological grades were Grade 3. Four patients accepted some cycles of gemcitabine-cisplatin (GC) treatment; these patients had a recurrence of bladder lesions or lesions remaining after GC treatment and then received DV combined immunization as neoadjuvant or bladder-sparing therapy.

Immunohistochemistry demonstrated that three cases were HER2 (2+), four cases were (3+), and the remaining two cases were (1+) and (0+), respectively. Roche VENTANA PD-L1 (SP263) tests all resulted in a combined positive score of <1%. Of these, three cases presented with underlying comorbidities, including three with hypertension, one with diabetes, and one with a lower risk of prostate cancer.

The median follow-up duration for these patients was 12.0 months (range from 8.0 to 17.0 months), calculated from the initiation of combination therapy to the end of follow-up.

### 3.2 Treatment and efficacy

All patients underwent treatment with DV combined with PD-1 inhibitors. This included six patients who received three courses of combined therapy, two patients who underwent four or more courses of treatment, and one patient who underwent two courses of treatment. Regarding the choice of PD-1 inhibitors, five patients were treated with tislelizumab 200 mg every 3 weeks (Q3W), and four patients were treated with toripalimab 3 mg/kg every 2 weeks (Q2W) (Table 1).

**FIGURE 1:**
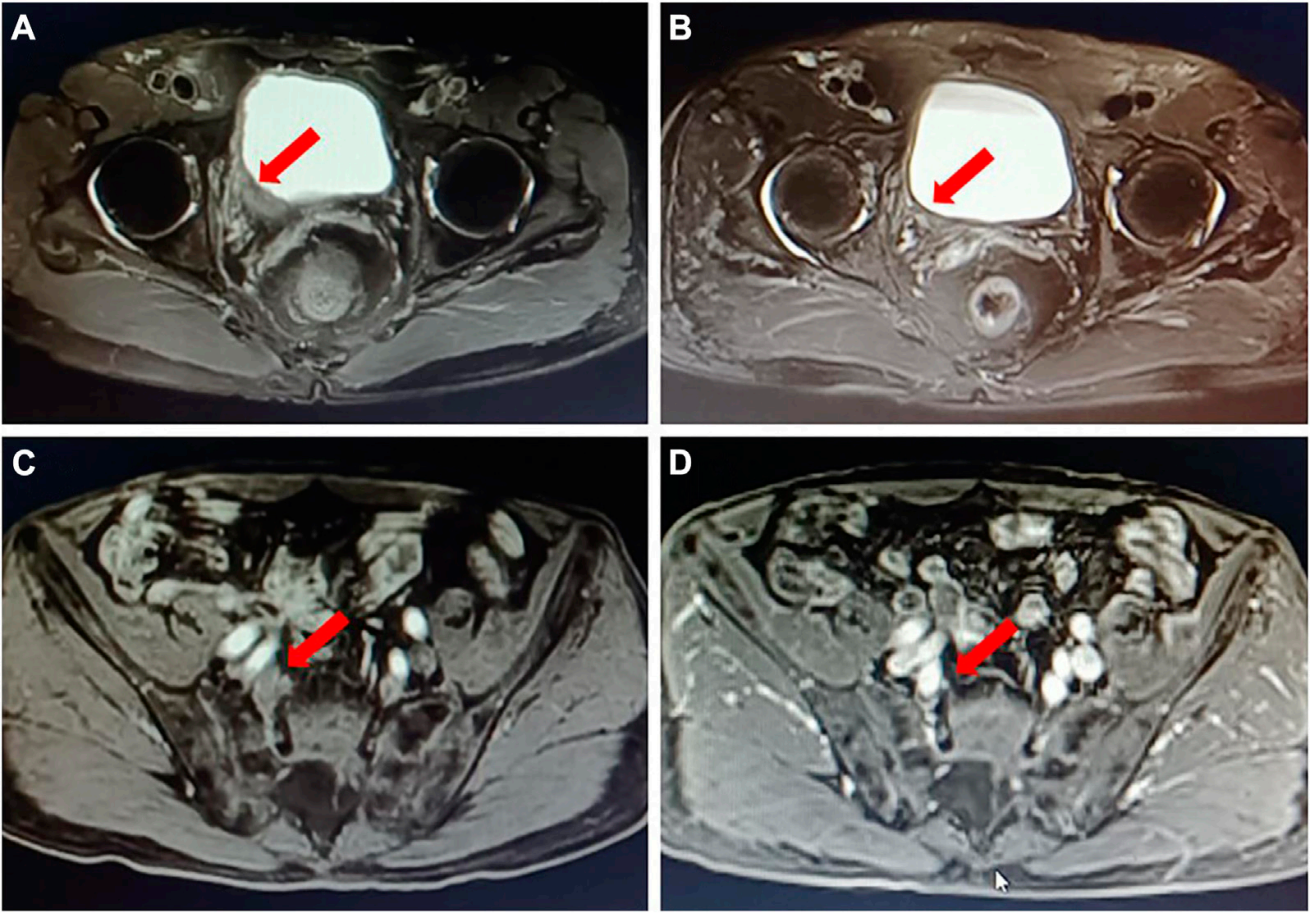
A typical case of neoadjuvant therapy of DV in combination with an immune checkpoint inhibitor. A 72 years old male patient with “gross hematuria” was admitted; MRI showed that a bladder tumor in the right wall of the bladder with the inner segment of the right ureteral bladder wall involved **(A)**; multiple mildly enlarged lymph nodes paravascular were found on the right iliac with a maximum of 1.1 cm, and lymph nodes metastasis were considered **(C)**. Pathology of biopsy confirmed bladder high-grade urothelial carcinoma, with PD-L1 low expression (IHC) and HER2 (2+) (IHC). DV 2 mg/kg plus toripalimab 3 mg/kg, Q3w was given as neoadjuvant therapy for three circles. Then MRI was performed to evaluate the outcomes of the neoadjuvant treatment; it showed a radiological complete response **(B, D)**. After laparoscopic right pelvic lymph node dissection with partial bladder incision and right ureteral bladder replantation, the postoperative pathology confirmed no evidence of cancer, suggesting a pathological complete response.

The treatment efficacy was finally evaluated by comparing imaging changes, urine cytological alterations, and results from transurethral resection. An ORR was achieved in eight cases, which included CR in five cases (Figure 1) and PR in three cases. rCR was entirely consistent with pCR.

By the end of the follow-up period, the median rPFS of these patients was 12.0 months (range: 8.0–17.0 months) (Table 2). One patient with HER2 (0+) was diagnosed with PD and underwent radical cystectomy; the pathological stage progressed from ypT2N0M0 to ypT3aN2M0. The treatment was then switched to GC combined with radiotherapy as adjuvant chemotherapy and primary radiotherapy. No tumor recurrence or metastasis was observed at the 9-month follow-up.

**TABLE 2:** Assessment of treatment efficacy.

Valuable			Numbers
ORR	CR	rCR	(*n* = 5)
—	—	pCR	(*n* = 5)
—	PR	rPR	(*n* = 3)
—	—	pPR	(*n* = 3)
—	SD	rSD	0
—	—	pSD	(*n* = 1)
—	PD	rPD	(*n* = 1)
—	—	pPD	0
rPFS (m.)	Range	8.0–17.0	—
—	medium	12.0	—

### 3.3 Advance effects and classification

In general, the incidence and severity of complications were manageable in these patients, and no grade 4 or 5 adverse events were observed. Treatment-related adverse reactions were reported in eight patients. The most observed symptoms included loss of appetite, rash, and fatigue, each occurring in three cases, all classified as grade I or II.

Additional adverse reactions such as hypothyroidism, fatigue, abnormal liver function, and peripheral sensory neuropathy were reported in two cases, grade I or II. A single instance of each of the following was reported: immune pneumonia (grade I), abdominal pain (grade I), nausea (grade II), joint congestion (grade II), gastrointestinal bleeding (grade III), and intestinal obstruction (grade III) (Table 3).

**TABLE 3:** Adverse reactions and grading.

Treatment-related adverse reactions	Any grade(N, %)	Grade Ⅰ-Ⅱ (N, %)	Grade Ⅲ-Ⅳ (N, %)
Any event	8 (88.9)	8 (88.9)	2 (22.2)
Decreased appetite	3 (33.3)	3 (33.3)	—
Rash	3 (33.3)	3 (33.3)	—
Weak	3 (33.3)	3 (33.3)	—
Hypothyroidism	2 (22.2)	2 (22.2)	—
Fatigue	2 (22.2)	2 (22.2)	—
Abnormal liver function	2 (22.2)	2 (22.2)	—
Peripheral sensory neuropathy	2 (22.2)	2 (22.2)	—
Immune pneumonia	1 (11.1)	1 (11.1)	—
Abdominal pain	1 (11.1)	1 (11.1)	—
Nausea	1 (11.1)	1 (11.1)	—
Joint congestion	1 (11.1)	1 (11.1)	—
Gastrointestinal bleeding	1 (11.1)	—	1 (11.1)
Intestinal obstruction	1 (11.1)	—	1 (11.1)

## 4 Discussion

This real-world study explores the application of DV combined with PD-1 inhibitors in locally advanced bladder urothelial carcinoma. In this study, DV combined with either tislelizumab or toripalimab demonstrated promising responses in patients with locally advanced urothelial carcinoma. The study enrolled nine patients with locally advanced urothelial carcinoma, of which five achieved confirmed CR and three achieved PR, resulting in an ORR of 88.9%. rCR was entirely consistent with pCR. The median rPFS was 12.0 months (range: 8.0–17.0 months).

An open-label, single-arm, multicenter phase Ib/II clinical trial demonstrated that four patients with locally advanced urothelial carcinoma and HER2 IHC 1+/2+/3+ achieved a cCR rate of 100% after receiving DV plus tislelizumab as neoadjuvant therapy (Wen, 2022). However, our study involved a more diverse patient population: nine patients, including three recurrent cases and one with HER2 (0). In another retrospective study, seven patients with HER2 overexpressing (IHC 2+ or 3+) NMIBC, who could neither have their bladder tumor completely resected nor tolerate surgery, were treated with either DV or a combination of DV and ICIs, showing promising efficacy and an ORR of 85.7% for all patients (Hu et al., 2023). The patient populations in these studies were distinct; the previous study enrolled all NMIBC patients, while our study focused on MIBC, which is associated with poorer survival outcomes for patients with urothelial carcinoma. Overall, this retrospective study represents a successful exploration of neoadjuvant or bladder-preserving therapy in the real-world setting using DV combined with immunotherapy.

Besides short-term efficacy, the safety of DV combined with PD-1 inhibitors during therapy in patients with MIBC is of utmost importance. In this study, no adverse events (AEs) of grades 4 or 5 were observed, with grade 1 or 2 AEs accounting for 88.9% of the total. Anorexia, rash, and fatigue were the most frequently reported AEs. Importantly, these events were of grade 1 or 2 severity, transient, and could be effectively managed in the outpatient setting without necessitating any dose modification or interruption. No new AEs were reported, and all observed AEs were manageable. Furthermore, there were no recorded fatalities during the therapy period.

An essential finding of this study was the anti-tumor activity of DV combined with PD-1 inhibitors in patients with HER2 IHC1+, IHC 0, and PD-L1< 1%. This effect is not solely due to the nonspecific bystander effects of the cytotoxic drugs released by DV. Our data strongly support a T cell-dependent mechanism and the efficacy of the combination therapy. This effect is achieved through non-redundant yet complementary mechanisms. Specifically, DV enhances T cell infiltration into the tumor by inducing tumor-specific, adaptive anti-tumor immunity, while PD-1 blockade rejuvenates exhausted T cells (Huang et al., 2022). Other chemotherapy regimens have had similar therapeutic benefits (Haratani et al., 2020; D’Amico et al., 2019; Wang et al., 2018).

Some limitations to our study should be acknowledged. The most significant was the lack of a mature follow-up period to provide survival outcomes. Extended follow-up was required, particularly focusing on long-term bladder preservation and survival. Another limitation was the small sample size of patients with cT2-T3bN0-2M0 tumors included in our study; we will embark on a prospective, case-control multicenter study, including evaluating the efficacy of the combination therapy in specific subgroups based on HER2 expression levels, to further validate our findings and provide new approaches to guide neoadjuvant therapy or bladder-sparing treatment for bladder cancer. Furthermore, we could not obtain data on survival rates with our limited observation period and sample size; we will include this subset of patients to observe survival data in future studies.

The promising safety profile and anti-tumor activity demonstrated by the combination of DV and PD-1 inhibitors in this real-world study lay a solid foundation for a phase 2 clinical trial exploring DV in combination with PD-1 inhibitors in patients with MIBC. Our study data were the most extended median follow-up time for DV plus immunotherapy to date, and our findings provided a new possibility for DV plus immunotherapy as neoadjuvant therapy or treatment of sparing bladder.

## 5 Conclusion

The combination of DV and PD-1 inhibitors demonstrated significant improvements in ORR in patients with MIBC across all HER2 IHC levels (0/1+/2+/3+). This regimen also reported the longest median rPFS to date, solidifying the combination of DV and PD-1 inhibitors as a compelling treatment choice for patients with T2-T4aN0-3M0 staging. Furthermore, this study confirms that the combination treatment maintains a manageable safety profile even with extended treatment duration.

## Data Availability

The original contributions presented in the study are included in the article/Supplementary material, further inquiries can be directed to the corresponding author.
